# Supplementary material for: Daily reference evapotranspiration prediction for irrigation scheduling decisions based on the hybrid PSO-LSTM model
Source: PLoS One. 2023 Apr 18;18(4):e0281478. doi: 10.1371/journal.pone.0281478 (PMC10112815; doi:10.1371/journal.pone.0281478)
Supplement: Attachment — Submitted filename: Response to reviewers.docx [file pone.0281478.s008.docx]

Dear reviewer,

Thank you for your giving us an opportunity to revise our manuscript!

It can be found that the prediction accuracy of the optimized model in the revised manuscript is lower than the prediction accuracy of the model in the original manuscript. The main reason is that the model is overtrained in the original manuscript. Conversely, the optimized model in the revised manuscript is not overfitted.

Reviewer #1: This paper presents a hybrid approach for predicting evapotranspiration levels. While the approach (producing a hybrid solver) is not particularly novel, the results themselves have some value. There are a number of ways in which this manuscript can be improved. These are detailed below and categorised as minor and major changes.

Minor:

* Abstract: This is far too long and in places has far too much detail. Lines 14-21 (manuscript line numbers) have far too much methodological detail and need to be summarised into just a couple of sentences.

* There are a few English expression issues through the manuscript. It needs to be proofed before the next revision.

* You need to use consistent math typesetting throughout the manuscript. One of the biggest issues is that variables are inconsistently italicised. Italicised variables tell the reader that an item is a variable. For example, in lines 129-148 no italicisation is present, however, in lines 308-315 it is. You need to carefully check the entire manuscript to make sure your mathematical representations are uniform.

* Lines 105-111: You refer to Section numbers in these lines, however, each section does not have a number, only a name. I suggest you just name the sections in these lines. Also, it makes no sense at this point to talk about what is in Section 1, as we are now at the end of Section 1. Remove this.

* Line 268: A reference is needed after “Fuller test”.

* Line 275: Do not put a space between “95” and “%”. Simply have “95%”. This occurs frequently throughout the manuscript and needs to be changed in all places.

* Line 290: Add a reference for the “Keras framework”.

* Table 2 is presently unnecessary as you do not talk about runtime as part of your results. This will also be discussed in the “Major” section below.

* Line 299: What do you mean by “preliminary experience”? Describe exactly what this preliminary experience entailed. I.e., what steps did you take to derive the bounds of 10 and 60?

* Line 439: “range” not “rang”.

* Line 462: “Table 6” not “table 6”.

* Line 495: The first five words in this line have no spaces between them. Add the spaces.

* Line 516: “A PSO algorithm” not “An PSO algorithm”.

Response: The above minor revisions have been completed in the revised manuscript.

Major

* Lines 113-121: It would be preferable to study the data from more than one site so that you can compare results and produce more robust results at the end. The results from one site only are not generalisable.

Response: Three weather stations (Fengxiang, Xianyang and Pucheng, all three sites are located in the Guanzhong Plain) were selected as the new study sites, and the optimized hybrid model were training and tested at those weather stations.

* There is no rationale why the PSO algorithm is chosen to hybridise with LSTM. Perhaps there are better algorithms? Some discussion of this point is necessary.

Response: The PSO is an evolutionary computational technique that originated as a simplified model of the population intelligence algorithm used in a study on the foraging behavior of birds. The algorithm has the advantages of a simple structure, easy implementation, and a strong global capability, which seeks the optimal value of the complex space through information transmission and competition between individuals.

* Lines 280-281: Why were the data split this way, i.e, different time periods? There may have been different conditions at each of those time periods, making training and validation data sets somewhat incompatible. Why not split it where both sets have the same time period (1980-2018) and use half the data each?

Response: Daily ETo sequence data from the study site were recorded from 1980 to 2019, over the 40-year period in question, there have been marked changes in global ambient temperature and other weather data. In this study, the ET_o_ was calculated by mixing six weather parameters, it can be seen from Table 1 that the standard deviation of the ET_o_ is much smaller than the standard deviation of five weather parameters at each station. Therefore, this paper ignores the influence of meteorological parameter changes on the prediction model. The data were split into training set (1980-2007), test set (2008-2018) and prediction set (2019), the data number of training set, test set and prediction set were 10227, 4021, and 365, respectively.

* As you have a hybrid algorithm, i.e., two algorithms are run and co-operate with one another, however, this always incurs a large computational (runtime) cost. Computational times are not reported in this paper (see comment in “Minor” section). How does your hybrid algorithm compare in terms of computational time to the algorithms you report in Table 6? Also, what is the comparison between the two algorithms in your hybrid? What resourcing do each of these required compared to the other? How many iterations are each run for? I see you have 20 for PSO in Line 303, but what are all of the other parameter values and why did you choose the values you used?

Response: The computational time is shown in the revise manuscript.

According the comment of Reviewer 2: The comparison against other techniques for this prediction problem is fatally flawed by comparing reported performance on _different_ datasets. Either rerun your technique on those other datasets or rerun those other approaches on yours (or even do both), but no inferences can be drawn from the current analysis.

Give the above comments, the table 6 was deleted in the revised manuscript, two hybrid model were optimized using the PSO, During the optimization process, the two models obtained the prediction 400 and 180 results under different hyperparameters combinations, respectively. Due to the long iteration time, the hybrid model unable to training more iterations in a short time. The training time and fitness of two model for each iteration also shown in Fig 7 and Fig 11.

Reviewer #2: The paper presents a combination of PSO for selecting values for two hyperparameters for an LSTM ANN for predicting reference evapotranspiration based on meteorological data based on a long time series for a single site. The authors have clearly devoted considerable effort to this, and have in parts conducted rigorous evaluation of the predictor's capabilities. However, there are two major methodological flaws that require the work be reconsidered outside of the review process:

1. The work's stated contribution is in the use of PSO to tune a subset of hyperparameters for LSTM, yet it is not demonstrated why PSO is suitable for this task, when the defined search space is so small (2,550 alternatives). A grid search at different levels of granularity would likely be just as effective at finding good hyperparameter values for equivalent computational effort. (Indeed, given the work only involved three [if I understand correctly] randomised trials the same effort could be used to explore over 45% of the entire search space).

Response: Firstly, The PSO is an evolutionary computational technique that originated as a simplified model of the population intelligence algorithm used in a study on the foraging behavior of birds. The algorithm has the advantages of a simple structure, easy implementation, and a strong global capability, which seeks the optimal value of the complex space through information transmission and competition between individuals. Which shows that the PSO can used to optimization parameters.

During the optimization process, the two models obtained the prediction 400 and 180 results under different hyperparameters combinations, respectively. Due to the long iteration time, the hybrid model unable to training more iterations in a short time. The training time and fitness of two model for each iteration also shown in Fig 7 and Fig 11.

One of the most common approach for optimizing the hyperparameters is through grid search. However, grid search suffers the curse of dimensionality when the number of hyperparameters are very large. In fact, many swarm intelligence algorithms were used to optimize the hyperparameters of deep learning network, more and more relevant research literature are published.

2. The comparison against other techniques for this prediction problem is fatally flawed by comparing reported performance on _different_ datasets. Either rerun your technique on those other datasets or rerun those other approaches on yours (or even do both), but no inferences can be drawn from the current analysis.

Response: Give the above comments, the table 6 was deleted in the revised manuscript, two hybrid model were optimized using the PSO, During the optimization process, the two models obtained the prediction 400 and 180 results under different hyperparameters combinations, respectively.

In order to evaluate the performance of the optimized model at other three stations, the optimized model was evaluated using the data from other three stations. At the same time, in order to verify the accuracy of the optimized model under different period, four types of periods are divided, including type A (training set 1980-2007,10220, test set 2008-2018, 4015), type B (training set 1980-2017,13869, test set, 1981-2018,13869), type C (training set 2003-2014, 4380, test set, 2015-2018,1460) and type D (training set 2011-2017, 2190, test set, 2017-2018, 730). The amount of data for each type of training set and test set is shown in the (Fig. 6).

Additional comments, mostly in paper order, follow:

PLOS ONE has a very broad audience, so explaining transpiration and evapotranspiration very early in the paper is required. Not everyone will remember their high school biology.

Response: Reference evapotranspiration (ETo) is one of the main factors in agricultural production and hydrological cycles, which plays a broad and important role in agricultural irrigation decision-making, hydrological prediction and scheduling, crop growth simulation, and climate disaster monitoring, and its precise prediction is important for the rational use and management of water resources

The opening literature review covers a lot but doesn't say much other than that other approaches focus on daily prediction instead of forecasting. I suggest expanding the analysis to better highlight what is novel in your approach.

Response: The decision making in irrigation scheduling depends on forecasts of 1 to 10 days ahead, which is critically important to determine crop water requirements and real time irrigation scheduling. The shorter time from daily to 7 days can be useful in planning the use of irrigation systems as well as in optimizing system power consumption. The innovation of this paper lies mainly in the following: two hybrid deep neural network model were proposed for daily ETo forecasting. the number of hidden neurons, dropout and look-back in LSTM are optimized by PSO. The optimized models were tested with four different datasets, and the proposed models achieved good results in forecast accuracy under different datasets.

Description of the approach:

- The components of Figure 3 should be explained in the text. Does it represent a single neuron, or the smallest collection neurons, etc.? Currently a lot of the explanation in the section "Long short-term memory network model" has insufficient detail except for those already familiar with it

Response: The components of Figure 3 represent a single neuron, according to the comment of editor, and the limitations of the reproduction ratio of scientific papers, it is difficult to extended relevant knowledge.

- In addition to the detailed explanation given for PSO it would be helpful to describe the communication topology used: this appears to be a gbest PSO (or PSO with a fully-connected topology)

Response: Equation 10 and Equation 11 describe the communication mechanism of the PSO. According to the comment of editor, the limitations of the reproduction ratio of scientific papers, it is difficult to extended relevant knowledge.

- The gbest PSO is very fast to converge (to local optima). Was this a deliberate design choice? Explain why this version of PSO was chosen

Response: Due to the long iteration time, the hybrid model unable to training more iterations in a short time.

- Another figure illustrating the effect of the different hyperparameters that are controlled by the PSO would greatly help in the explanation of the hybrid system

Additional detail of the hybrid optimisation method is required when it is first described (near the 8 steps):

Response: the framework of two PSO-LSTM model is constructed, as shown in (Fig. 3).

**Step 1**: Import the dataset and normalization, and dividing the dataset into training set, validation set and test set.

**Step 2**: PSO is used to optimize the hyperparameters.

1) Parameter initialization. The particle dimension, population size, iterations, learning factors c_1_ and c_2_, inertia weight w, velocity and position are determined.
2) Initialize the velocity and position of particles, and randomly generate population particles.
3) The mean absolute error is taken as the fitness value of the model, and the pbest and gbest are calculated with the fitness value of the particle.
4) After each iteration, the position and velocity are updated, and the fitness is also calculated, at the same time, the pbest and gbest are also updated.
5) Determine whether the termination condition is met. If satisfied, the optimization results were obtained. Otherwise, go back to step 3).

**Step 3:** The optimized LSTM model were obtained, and training and evaluate the optimized model with other weather stations data.

- Was each PSO particle really only optimising a 2D vector? (See my summary comment (1) at the top)

Response: for the first hybrid model, the PSO particle was optimized a 2D vector, and for the second hybrid model, the PSO particle was optimized a 4D vector.

- The description of the hybrid model implies that the entire population of PSO solutions is used to define just one LSTM network. Presumably that is _not_ the case, but the description currently doesn't make that clear. If it _is_ the case that the population of solutions is used to train a single LSTM network then clearly explain how that is done

Response: In this study, the framework of two PSO-LSTM model is constructed, as shown in (Fig. 3). the first model consisting of a LSTM layer, a flatten layer and a dense layer, and two important hyperparameters, the number of the first hidden layer neurons (N_1_) and the look back (time windows, T_1_) were used to be optimized. The second model consisting of two LSTM layer, a flatten layer and a dense layer, and four important hyperparameters, the number of the first hidden layer neurons (N_2_), the second hidden layer neurons (N_3_), the dropout (D) and the look back (T_2_) were used to be optimized. Those hyperparameters are regarded as the particles in PSO. The mean absolute error (MAE) of the model’s predicted and actual values are taken as the fitness function.

- Overall, the 8 steps shown before Figure 4 would be better formatted as an algorithm so that the parts that repeated are clearly distinct from those steps that occur only once at the start or end of the overall process

Response: In this study, **Step 1**: Import the dataset and normalization, and dividing the dataset into training set, validation set and test set.

**Step 2**: PSO is used to optimize the hyperparameters.

6) Parameter initialization. The particle dimension, population size, iterations, learning factors c_1_ and c_2_, inertia weight w, velocity and position are determined.
7) Initialize the velocity and position of particles, and randomly generate population particles.
8) The mean absolute error is taken as the fitness value of the model, and the pbest and gbest are calculated with the fitness value of the particle.
9) After each iteration, the position and velocity are updated, and the fitness is also calculated, at the same time, the pbest and gbest are also updated.
10) Determine whether the termination condition is met. If satisfied, the optimization results were obtained. Otherwise, go back to step 3).

**Step 3:** The optimized LSTM model were obtained, and training and evaluate the optimized model with other weather stations data.

Regarding the data splits: although contiguous time periods are required for time series regression, is it valid to split the data into time periods that occurred under different climatic conditions? There are clear changes in global ambient temperatures and other meteorological data over the 40-year period in question. While predicting ETo from meteorological data should work for any inputs, splitting the data this way will likely mean training under one set of conditions and then evaluating the model's efficacy under different conditions.

Response: Daily ETo sequence data from the study site were recorded from 1980 to 2019, over the 40-year period in question, there have been marked changes in global ambient temperature and other weather data. In this study, the ET_o_ was calculated by mixing six weather parameters, it can be seen from Table 1 that the standard deviation of the ET_o_ is much smaller than the standard deviation of five weather parameters at each station. Therefore, this paper ignores the influence of meteorological parameter changes on the prediction model. The data were split into training set (1980-2007), test set (2008-2018) and prediction set (2019), the data number of training set, test set and prediction set were 10227, 4021, and 365, respectively.

PLOS ONE is a generalist scientific journal, so please include footnotes to explain/link to pandas and keras. (The majority of readers will not know these are Python libraries or what they are for.)

Response: ([https://keras.io/) and](https://keras.io/)%20and) reference [35]

How was each PSO solution's 'fitness value' determined given the four evaluation measures? Was just one used or were they combined in some way?

Response: The mean absolute error (MAE) of the model’s predicted and actual values are taken as the fitness function.

Issues with the evaluation of the hybrid system:

- Was the hybrid system only run for just three randomised trials? Given the likely variability in such a complex system's behaviour this should be increased to at least 10 to get some representative indication of performance.

Response: Due to the long iteration time, the hybrid model unable to training more iterations in a short time.

- However, given the solution space is 51 (node count range) x 50 (look back range) = 2,550 and each run explores up to 400 (20 solutions x 20 iterations) hyperparameter combinations, which is ~15.5% of the search space, is the PSO really worthwhile? Merely evaluating the system's performance properly with 10 randomised trials would involve the same effort as evaluating 150% of the entire search space. So: is the hybrid system bring presented as a viable alternative for larger problems?

Response: Due to the long iteration time, the hybrid system is unreliable as the a viable alternative for larger problems.

- And: why was a random search of that same search space (also for 400 solutions) not examined to judge the contribution of the PSO search?

Response: For the first optimized model, the PSO-LSTM optimization iterated 20 times. (Fig. 7) present the training time, maximum fitness, average fitness and minimum fitness in each iteration. From the figures, we can find that the training time cost increases rapidly with the number of iterations. Although the batch size=64, T1 and N1 of hybrid model is [1,50] and [10, 60], respectively, the utilization of CPU gradually decreases during the training process. The training time for the first and last iterations is 4.27h and 17.30h, respectively, and the total training time of 20 times iterations is 221.25h (9.21 days). The fitness gradually decreases as the number of iterations increases, and the minimum fitness is much smaller than the maximum fitness of each iteration. After 20 times iterations, the maximum, average and minimum fitness of optimal particle are 0.637, 0.608 and 0.597, respectively. After 20 times iterations, the hyperparameter combination corresponding to the minimum fitness is [5, 24], it means that the look back =5, the number of first layer neurons=24 is the optimal combination for the first model.

For the second optimized model, the PSO-LSTM optimization iterated 9 times. (Fig. 11) present the training time, maximum, average and minimum fitness in each iteration. From the figures, we can find that the training time increases rapidly with the number of iterations. Although the batch size=64, the T2, N2, and N3 of hybrid model is [1,200], [1,200], and [1, 200], respectively, and D=0.1,0.2,0.3, the utilization of CPU gradually decreases during the training process. The training time for the first and last iterations is 18.20h and 147.84h, respectively, and the total training time of 9 times iterations is 806.03h (33.58 days). The fitness gradually decreases as the number of iterations increases, and the minimum fitness is much smaller than the maximum fitness of each iteration. After 9 times iterations, the maximum, average and minimum are 0.619, 0.606 and 0.600, respectively. After 9 times iterations, the hyperparameter combination of the optimized model corresponding to the minimum fitness is [22, 175, 39, 0.2], it means that the look back=22, the number of first layer neurons =175, the number of second layer neurons =39, and dropout=0.2 is the optimal combination for the model.

It is not clear how the contents of Table 4 were derived. Are these a collection of the best solutions discovered by PSO?

Figure 6 (ETo predictions and true values across 11 years) is of limited utility since it shows not much more than what the numerical accuracy measures did, that the LSTM model does quite a good job and gets it wrong sometimes. Even if the true value (plotted first) was entirely obscured by the predicted value (plotted over the top) then this wouldn't necessarily confirm that the learned model was good, only that its predictions lie somewhere behind the many points plotted over the top.

This text following Figure 6 makes no sense as it describes the 'value range of the prediction residual' twice: The prediction residual of optimized best model for validation set was 376 shown in (Fig 7), it can be seen from the figure that the value range of the 377 prediction residual was from -2.0 to 2.0 mm, and the value range of the 378 prediction residual was from -0.5 to 0.5 mm."

Figure 8: While showing a line of best fit reinforces the linear relationship between predicted and actual values, showing the line representing a perfect relationship would be more meaningful as it reveals that the predictions tend to underestimate higher actual values of ETo, which does not appear to be discussed in the paper.

It is good that the results in Figure 10 are explained in the text, because it is actually not possible to clearly see when the model is predicting higher than ground truth or lower than ground truth. This is because it is difficult to match each predicted point to its associated ground truth point: only some vague trends can be seen. I don't have a good suggestion for this other than the scatter plots you already include of predicted versus actual.

As with Figure 8, Figure 12 would be more informative is the perfect fit line were shown instead, revealing that the model predicts lower for larger ground truth values.

Table 6 should use the same number of significant digits for all entries. And why are the MAE and MSE entries for SVR-RBF not bolded, since they are the same as PSO-LSTM?

Most problematic, why are performance of other models reported for different data within Table 6 (see, e.g., SVR-RBF, RF, CNN and LSTM, which were evaluated for a different location, in Portugal, similarly for the work of Ferreira, which reports performance on data from Brazil, and Farooque, who studied data from PEI [which definitely does _not_ have a monsoonal pattern like the Wugong site you have based your work on]). Such a comparison is completely inappropriate. Those alternative models should be applied to the same training and testing data as used in your work in order to create a valid comparison, or you should apply your approach to those other datasets, or both.

Response: Table 4, Table 5, Table 6, Figure 6, Figure 7, Figure 8, Figure 9, Figure 10, Figure 11, and Figure 12 were deleted in the revised manuscript.

Subset of minor grammatical issues:

- Abstract: opening sentence should be split after irrigation,

- Split the later sentence between "decision, many"

- Inconsistent ET_o formatting (should be fixed during final typesetting)

- Intro, paragraph 1: The final sentence should be split between ET_o and this method

- There are other similar grammatical errors but I will not list any more

- "numbers of" is used where "number of" should be in relation to the number of hidden layers

- Use capital S Section when referring to Section 1, etc.

- In the explanations of the variables in (1) "represent" should be "represents" (third person singular)

- typo line 431 reads 2018 instead of 2019

- typo line 439: rang --> range

Response: The above minor revisions have been completed in the revised manuscript.

All authors have read and approved the re-submission of the manuscript!

If you have any question, please let me know!

Thank you for your consideration of our paper and we are looking forward to hearing from you!

Sincerely, Weibing Jia，jiawb02@stu.xjtu.edu.cn

PhD student，The State Key Laboratory for Manufacturing Systems Engineering, Xi’an Jiaotong University，Xi’an 710049, China
