# Supplementary material for: Daily reference evapotranspiration prediction for irrigation scheduling decisions based on the hybrid PSO-LSTM model
Source: PLoS One. 2023 Apr 18;18(4):e0281478. doi: 10.1371/journal.pone.0281478 (PMC10112815; doi:10.1371/journal.pone.0281478)
Supplement: Attachment — Submitted filename: Response to reviewers.docx [file pone.0281478.s009.docx]

Dear reviewer,

Thank you for your giving us an opportunity to revise our manuscript again!

Reviewer #2: I'd like to thank the authors for responding so positively to the critiques of the original submission. As with that original submission it is clear that a significant amount of researcher and computer time has been invested in the work. The expanded number of datasets and alternative ways they have been split to create different training and testing combinations is a really welcome change to the study.

There remain two issues with the work, one of which has not been addressed from the original reviews.

1. While it's good that the runtime is now given (see major point 2 below), and in part this explains the low number of solutions produced by the solver and, the question remains: why PSO? No arguments are put forward to explain why such a metaheuristic will give better search performance than some other (simpler) alternative, and certainly no experimental results are included to demonstrate this either. Some specific issues:

Response: in order to compare the search performance of metaheuristic and simpler alternative, the grid search method were used to optimize the hyper parameters of the two LSTM models, the grid search schemes as show in Table 1. For the first LSTM model, the look back (T_1_) is increase from 12 to 50, at the same time, the first hidden layer neurons (N_1_) is increase from 45 to 60, a total of 608 hyperparameters combinations were trained, and the hyperparameter combination corresponding to the minimum fitness is [13, 52], it means that the look back =13, the number of first layer neurons=52 is the optimal combination for the first model.

For the second LSTM model, the look back (T_2_) is 5, 50, 100, 150, and 200, respectively. The first and second hidden layer neurons (N_2_ and N_3_) are increase from 5 to 200 by 5 each time, a total of 9000 hyperparameters combinations were trained, and the hyperparameter combination corresponding to the minimum fitness is [200, 140, 170, 0.1], it means that the look back are 200, the number of first and second layer neurons are 140 and 170, respectively, and the Dropout are 0.1 are the optimal combination for the second model.

Table 1. The two grid search schemes for two model parameters.

| Model | Look back | First hidden neurons | Second hidden neurons | Number of trained models |
| --- | --- | --- | --- | --- |
| #1 | [12,13,14,···,50] | [45, 46, 47,  ···,60] | / | 608 |
| #2 | [5,50,100,150,200] | [5, 10, 15,  ···,200] | [5, 10, 15,  ···,200] | 9000 |

The table 2 shows the results of comparison of minimum fitness and cost time between grid search and PSO-LSTM. For the first model, the minimum fitness of two methods are 0.595 and 0.597, respectively, the trained time are 58.33h and 221.25h, respectively. It can be seen that the difference between the optimal minimum fitness of the two methods is small, and the cost time of grid search method is far less than that of the PSO method. This means that for the first LSTM model, the grid search method is a better model hyper parameters optimization method.

For the second model, the minimum fitness of two methods are 0.593 and 0.600, respectively, the trained time are 4826.94h and 806.03h, respectively. It can be seen that the difference between the optimal minimum fitness of the two methods is also small, and the cost time of PSO method is far less than that of the grid search method. This means that for the second LSTM model, the PSO method is a better model hyper parameters optimization method.

Table 2. Comparison of minimum fitness and cost time between Grid search and PSO-LSTM.

| Model | Minimum fitness (MAE) | | Cost time (h) | |
| --- | --- | --- | --- | --- |
|  | Grid search | PSO | Grid search | PSO |
| #1 | 0.595 | 0.597 | 58.33 | 221.25 |
| #2 | 0.593 | 0.600 | 4826.94 | 806.03 |

In general, the grid search method is suitable for the LSTM model with few hyper parameters, for the LSTM models with multiple hyper parameters and a large range of hyper parameters, the training time cost of grid search method is very high. It can be seen that the PSO method can not only optimize multiple parameters in a relatively small amount of time, but also has a relatively high optimization accuracy, therefore, the PSO algorithm is more suitable for optimizing the LSTM model with multiple hyper parameters.

1.1 Figures 7 and 11 show that the minimum MAE of solutions at the end of the run is similar to the minimum at the start. Somewhat worringly, the minimum value is not monotonically decreasing, which should not be possible if particles' pbest solutions are shown. As in my original review: what has PSO added that random search could not achieve given equivalent computational effort?

Response: in this paper, the grid search method and PSO algorithm were used to optimized two type different LSTM models, and more than 10 000 models were trained and optimized. It is undeniable that the MAEs of those LSTM models have little difference. So the Figure 7 and 11 also show that the minimum MAE of solutions at the end of the run is similar to the minimum at the start. Considering the unpredictability of hyper parameters of the model, the minimum is not absolutely monotonically decreasing.

1.2 The long runtime notwithstanding the argument for using PSO has still not been made. The responses to reviewer comments do not justify its use (most merely re-explain this now very well-known algorithm) and instead repeatedly point out the long time to train the models. What evidence is there that (1) any form of metaheuristic search and (2) PSO in particular is more suitable for this problem than using some other technique (random search, grid search at varying granularities) with the same budget of 400 solution evaluations? Some attempt to address this question needs to be made. (Note that pointing to the improvement in solution quality over time does _not_ justify the use of PSO; because it is elitist the quality of each solution's pbest will be monotonically improving over time.)

1.3 (minor) It would still be worth explaining _why_ a gbest PSO was selected. This only requires a sentence and could just focus on the small number of iterations required by the long time to evaluate each solution.

Response: The PSO is the only form of metaheuristic search for the LSTM model in this paper, In fact, many swarm intelligence algorithms were used to optimize the hyperparameters of deep learning network, more and more relevant research literature are published. The Whale optimization Algorithm (WOA) and Grey Wolf Optimizer (GWO) were also used to optimized the LSTM model, and these two optimization algorithms also take a long time, and the optimization models are easy to fall into local optimization, so this paper dose not show the optimized result of these two models. If necessary, the optimization results of the these two models can also be provided.

One of the most common approach for optimizing the hyperparameters is through grid search. However, grid search suffers the curse of dimensionality when the number of hyperparameters are very large. Based on the reviewers’ suggestions, the grid search were also used to optimized the LSTM models, and 9000 LSTM models were trained, the best LSTM network structure has been selected, the results show that that the training time of the grid search is more longer, but the optimization results of the grid search algorithm are not significant. The random search algorithm has not been trained in the paper. The PSO algorithm has the advantages of a simple structure, easy implementation, and a strong global capability, which seeks the optimal value of the complex space through information transmission and competition between individuals.

In general, compared with the GWO, WOA and the grid search algorithms, the PSO algorithms is a relatively good algorithm.

2. It is good that the training time for the LSTM (hence, a measure of solution evaluation time) is now clearly stated, as this makes the number of iterations used much clearer. However, is this long runtime just an artefact of running it on an underpowered CPU (an i5 is only suitable for running office applications) and not a GPU as keras is intended? Using a more appropriate experimental machine would likely allow for more thorough (and far less time-consuming) experimental work.

Response: Thanks a lot for the reviewer’s suggestions. Using a more appropriate experimental machine would likely allow for more thorough experimental work, in order to achieve the comparability of different optimization methods, a more appropriate experimental machine was not used. To improve the training efficiency of the LSTM model, the grid search optimization LSTM were trained on four computers with the same configuration, and the grid search algorithm has been running on four computers for 4826.94h.

Other issues/comments:

- There is a conflict between the argument put forward for why the effect of climate change can be ignored in the data and the fact that alternative splits that use different time periods (past v future, future v past) have been used; the use of additional data splits is otherwise a really good inclusion in the work. The argument that begins line 364 begins by acknowledging the change in meteorological data over the 40 years, but it is nonsensical to compare the magnitude of the standard deviations of measures that use different units to argue that changes to the input features can be ignored. Further, comparing descriptive statistics over the entire time period cannot be used to dismiss observable differences within different contiguous subsets of the data. Is the argument starting at line 364 necessary given many of the experiments are made with different temporal splits? If you believe so then a _much_ stronger argument is required.

Response: the ET_o_ was calculated by mixing six weather parameters, and the change in meteorological data over the 40 years are obvious, therefore, the ET_o_ in the past 40 years has also changed. The research interval in this paper is 1 day, and the change of meteorological parameters in 1 day is random. This paper cannot accurately describe the change of meteorological parameters, so this paper ignores the influence of meteorological parameter changes on the prediction model. The data were split into training set (1980-2007), test set (2008-2018) and prediction set (2019), the data number of training set, test set and prediction set were 10227, 4021, and 365, respectively.

- Minor: the new text describing keras as 'an API designed for human beings, not machines' is marketing spin and does not belong in a scientific paper.

Response: the reference 35 were deleted, and revised to [35] Yulius Harjoseputro. A Classification Javanese Letters Model using a Convolutional Neural Network with KERAS Framework[J]. International Journal of Advanced Computer Science and Applications (IJACSA),2020,11(10)..10.14569/IJACSA.2020.0111014.

- In Section 2.1 Tables 2 and 4 are going to be difficult to format for the journal. However, it's also very difficult for the reader to interpret. Wouldn't these be better presented graphically?

Response: The format of Table 2, 3, 4 and 5 have been modified in the revised manuscript.

-The discussion of performance based on data split (around lines 548-9) is odd/uninformative. Since the different data splits correspond to training under one climate condition and testing on another, can something more be said about this outcome?

Response: the different data splits correspond to training under one climate condition, and in order to verify the accuracy of the optimized model under different period, four types of periods are divided, including type A (training set 1980-2007,10220, test set 2008-2018, 4015), type B (training set 1980-2017,13869, test set, 1981-2018,13869), type C (training set 2003-2014, 4380, test set, 2015-2018,1460) and type D (training set 2011-2017, 2190, test set, 2017-2018, 730). The optimized model were also trained and tested under four different datasets, it must be pointed out that the optimized model is retrained and tested with four different data sets again, instead of just using the optimization model to predict different test sets.

Minor typos noticed while reading:

- General: Please re-check the formatting after applying the changes, as some necessary spaces and text are missing and additional spaces have been inserted where they should not be.

- line 94: the edits have deleted the start of the sentence that used to say PSO optimizes...

- line 124: missing spaces after each of the place names and the opening (See general comment above as that is not the only problem with spacing in this revised text.

Response: The above minor revisions have been completed in the revised manuscript.

At the same time, the results of grid search method for two models were added in **the supporting information.**

All authors have read and approved the re-submission of the manuscript!

If you have any question, please let me know!

Thank you for your consideration of our paper and we are looking forward to hearing from you!

Sincerely, Weibing Jia，jiawb02@stu.xjtu.edu.cn

PhD student，The State Key Laboratory for Manufacturing Systems Engineering, Xi’an Jiaotong University，Xi’an 710049, China
